# Islamophobia in Canadian Nursing: An Interpretive Phenomenological Study Looking at the Experiences of Female Muslim Nurses

**DOI:** 10.1177/08445621261426504

**Published:** 2026-03-02

**Authors:** Zahra Upal, Jennifer Jackson

**Affiliations:** 1 2129 University of Calgary, Calgary, Canada

**Keywords:** Muslim nurses, Islamophobia, hijab, racism, critical race theory, interpretative phenomenological analysis

## Abstract

**Background:**

Muslim Nurses in Canada are experiencing Islamophobia in the workplace and in educational institutions. These experiences result in increased mental distress for these nurses, social isolation at the workplace, and considerations of leaving the nursing profession.

**Aim:**

This study explores the experiences of Islamophobia for Registered Nurses who wear the hijab at work in Canada. This study is grounded in Critical Race Theory and aims to answer the following research question: What are the experiences of Islamophobia for female Canadian Registered Nurses who wear the hijab? The participants in the study described experiences from their workplaces and their experience attending nursing school in Canada.

**Method:**

A total of six participants were interviewed, and the findings were analysed using the Interpretative Phenomenological Analysis method. Muslim nurses’ experiences were characterized by their sense of belonging.

**Findings:**

Key findings consist of the experiences of Islamophobia being related to the negative stereotypical identity of what it means to be a Muslim woman, being associated with these nurses, how they discover their own identity as nurses and finally, how they reconcile their personal and social identities as Muslim women with their identity as Registered Nurses.

**Conclusion:**

This study uncovers the process of self-discovery that Muslim nurses undergo after experiencing Islamophobia through being assigned an identity. This process enables them to reconcile what it means to be Muslim nurses, allowing them to practice comfortably at the workplace. Recommendations are that policy changes should be enacted, which protect Muslim nurses and work to prevent incidents of Islamophobia. Secondly, anti-racism training should be provided to both nursing students and staff to foster more supportive workplaces.

## Introduction and Background

Islamophobia amongst Muslim nurses in Canada is an issue that has not been significantly explored in the literature. As of 2021, there were 312,382 practicing Registered Nurses in Canada (Canadian Nurses Association, 2024). Although the exact number of Muslim nurses in Canada is currently unknown, what is known is that the Muslim population in Canada has been steadily growing. Therefore, it can likely be concluded that the number of Muslim nurses in Canada is also increasing.

Islamophobia is defined as: “any distinction, exclusion, or restriction towards, or preference against, Muslims (Runnymeade Trust, 2020, p. 1). After the terror attacks on the World Trade Center in the United States on 9/11, there has been a marked increase in Islamophobia experienced by Muslims (Clark & Saleh, 2019). From 2016 to 2021, Canada experienced the most significant number of Muslims killed in hate-motivated attacks of all the G7 countries (SenCA + Magazine, 2022). These data demonstrate that Islamophobia is still prevalent in Canada, 20 years after the events of 9/11. The attitudes that everyday Canadians hold regarding Muslims also affect Muslim nurses in this country.

A study conducted by authours Saleh et al. has found that Islamophobia is very much present in Canada's nursing profession, yet currently, there are limited data on this topic. A scoping review found that there is currently a lack of research on Islamophobia in healthcare, especially experiences of Islamophobia towards Muslim women (Camargo et al., 2023).

This study looks at the experiences of Islamophobia for female Muslim Registered Nurses who wear the hijab in Canada. The following research question was used to conduct this study: What are the experiences of Islamophobia for female Canadian Registered Nurses who wear the hijab? The participants in this study described experiences from their workplaces and from attending nursing school in Canada.

### The Concept of Othering as it Relates to Muslims

Muslims have also been subjected to a process called othering, defined as a process through which a group is marked as different and inferior because of skin colour, religion, gender, and thus alienating them through an “us versus them” mentality. (Curle, 2020). Consequently, othered persons may have restricted opportunities and be marginalized in society (Johnson et al., 2004). According to Clark and Saleh (2019) othering results in Canadian Muslims being perceived by non-Muslims as resisting the adoption of Canadian values (Environics Institute, 2016, p. 1).

Critical Race Theory (CRT) provides the core foundation for this study. CRT emphasizes that racism is ordinary, systemic, and sustained through long-standing structures of power rather than isolated incidents (Delgado & Stefancic, 2017). A key tenet of CRT, the social construction of race, suggests that racial categories are created and assigned by society, often through visible markers (Solórzano & Yosso, 2002).

This lens is especially useful for understanding Islamophobia, which scholars describe as a process of racialization. Garner and Selod (2015) explain that Muslims are racialized when beliefs, practices, or clothing are viewed as inherent traits and used to construct Muslims as a homogenous and inferior group. These socially invented assumptions, rather than any biological reality, shape how Muslims are perceived and treated.

For hijab-wearing Muslim nurses, the hijab becomes a highly visible signifier of “Muslimness,” making them more easily targeted by anti-Muslim attitudes (Chakraborti & Zempi, 2012). Research shows that these nurses are often associated with violence or extremism (Saleh et al., 2022), refused by patients and families (Kulwicki et al., 2008), and racialized regardless of their actual racial or ethnic background (Hampton & Hartman, 2019). Some also misinterpret the hijab as a symbol of oppression, leading to assumptions that Muslim nurses lack agency or competence (Saleh et al., 2022).

Framing these experiences through CRT helps clarify that the discrimination Muslim nurses face is not simply interpersonal bias, but part of broader racializing processes embedded within nursing. This study draws on CRT to understand how these systemic forces shape the everyday experiences of hijab-wearing female Muslim Registered Nurses and the unique forms of discrimination they encounter.

Interpretive Phenomenological Analysis (IPA) was selected as the methodological approach for this study. IPA is a qualitative method grounded in phenomenology that examines how individuals make sense of significant experiences in their lives. Developed in the 1990s, it explores how everyday events acquire meaning through reflection (Eatough & Smith, 2017; Smith et al., 2022). Through this approach, the researcher examines lived experience, engages in interpretive analysis, and examines each participant's account in depth before identifying connections across cases (Peat et al., 2019).

Given its emphasis on meaning-making, IPA is especially well-suited to this study. It enables a careful exploration of how Muslim nurses reflect on and interpret the everyday moments in which Islamophobia shapes their identities and their work as healthcare professionals.

## Design and Research Method

IPA draws on a phenomenological approach, making it well-suited to understanding how everyday experiences of Islamophobia become meaningful and significant for participants. In IPA, the participant is viewed as the experiential expert (Eatough & Smith, 2017). In this study, IPA allowed us to focus on how Muslim nurses interpret their experiences and the meanings they assign to them. Rather than treating experiences as fixed events, the IPA approach supported participants in reflecting on how they understood, processed, and made sense of Islamophobia over time.

The semi-structured interview design was central to supporting participant meaning-making. Open-ended questions encouraged participants to describe experiences in their own words. The follow-up prompts encouraged reflection on how these experiences affected their sense of identity, belonging and professional role. This approach allowed participants to pause, elaborate and revisit experiences, allowing meaning to emerge through narration rather than being imposed by the researcher. Participants were also invited to clarify or expand on earlier statements, thus allowing for deeper engagement with their experiences.

IPA's idiographic commitment also supported meaning-making by enabling an in-depth examination of each participant's account before identifying connections across participants. This process ensured that individual meanings were examined and noted before shared themes were developed, aligning with IPA's emphasis on understanding lived experience from the participant's perspective.

CRT provided the theoretical framework for this study, guiding both the design and analysis. The tenets of CRT informed key methodological choices. Ordinariness highlighted for us how Islamophobia operates in everyday nursing workplaces. Social construction emphasized how assumptions about Muslim nurses are socially created. Finally, narrative storytelling shaped the semi-structured interview design to allow participants to share their experiences fully. CRT also helped frame participants as a racialized group, recognizing that Muslim nurses, particularly those who wear the hijab, can experience discrimination based on both religious and racialized assumptions (Garner & Selod, 2015). This framework ensured that the study remained focused on understanding participants’ lived experiences within the broader structures of systemic inequity.

## Setting and Recruitment

This study was conducted in Canada and included six participants from Alberta and Ontario. The inclusion criteria for this study were female Registered Nurses, currently working in Canada, who identify as Muslim and wear a hijab at work. Participants were recruited via an invitation posted on the following social media platforms: X (formerly Twitter), LinkedIn, and Instagram. A total of nine people expressed interest in participating, of whom six met the inclusion criteria.

Data were collected through one-on-one, semi-structured interviews, which allowed us to focus on participants’ lived experiences while enabling narrative storytelling, a key tenet of CRT. The interviews were conducted, recorded, and transcribed via Zoom and lasted up to 90 min. Each began with a welcome and consent process, followed by demographic questions to capture participants’ age, work setting, and experience (see Table 1). Participants were then asked four open-ended questions about their experiences with discrimination and Islamophobia, with follow-up questions tailored to each conversation. The full interview guide is provided in Appendix A.

**Table 1.** Participant Demographics.

Demographic	Demographic Subcategories	(n)	Percent
Age			
	20–25	1	16%
	26–30	4	66%
	30+	1	16%
Ethnicity			
	South Asian	4	67%
	Black	1	17%
	South Asian/Arab	1	17%
Years of Experience			
	Less than 1 year	1	17%
	1-4 Years	3	50%
	5+ years	2	34%
Work Setting			
	Acute Care	3	50%
	Mental Health	1	17%
	Community	2	33%

This study has been inspired by the first authours own experiences with Islamophobia in Canada's nursing profession. To ensure reflexivity, she began by deeply analyzing her own personal experiences with Islamophobia. She reflected on how these experiences have impacted her as a nurse and, therefore, was better able to analyze whether she was attributing her own experience to the participants’. During the interview process, she made sure to stay present with the participant and attempted to deeply engage with what they were sharing, rather than focusing on her own experiences with Islamophobia.

During the data analysis process, lengthy discussions took place between the two authours of this study to ensure that the interpretation accurately reflected the participants’ experience. The authours also returned to the interview transcripts repeatedly to ensure that each quotation they interpreted considered the participants’ experience.

## Data Analysis

Data were analyzed using the seven-step process outlined in the IPA method (Smith et al., 2022). First, we listened to, transcribed, and re-read each interview multiple times to develop familiarity with the participant's account. We also made some notes on quotations to focus on later. In the second step, we took detailed notes which captured descriptive observations, shifts in tone, emotional emphasis, and early reflections on meaning. Consistent with IPA's interpretive focus, we paid attention to how participants made sense of their experiences and how these experiences shaped their judgments, actions, and identities. (Table 2)

**Table 2.** Steps of Data Analysis.

Step Number	What IPA says to do	What I did
1	Review transcript	Listen to audio recording and verified transcription was accurate. Made notes.
2	Creating exploratory notes	Create a detailed list of notes, made detailed observations.
3	Experiential statements	Created open codes followed by a search for connections between these codes to reflect the central tenets of CRT.
4	Connections amongst experiential statements	Charting the experiential statements to see which fit with each other and putting them into categories or themes.
5	Creation of Personal Experiential Themes (PET)	Naming the PETs and placing them in a table.
6	Creation of Group Experiential Themes (GETs)	Looking at the overall data including the relevant participant quotations, PETs and considering similarities and differences between them at a broader level to create the final themes (GETs).

In the third step, exploratory comments were transformed into experiential statements that captured key meanings within each transcript. Open codes were developed and examined for conceptual connections, while the tenets of CRT, especially social construction and interest convergence, guided our attention to how racism and power operated within participants’ accounts. CRT supported interpretation by highlighting how assumptions about Muslim nurses’ competence are socially constructed, and how power relations within nursing shape the conditions under which discrimination occurs.

The fourth step involved charting the experiential statements to see which fit together and placing them into categories (Smith et al., 2022). The experiential statements in the transcript were examined to identify similarities among them. Based on this, we organized the statements into themes.

In line with IPA's idiographic commitment, the fifth stage consisted of us analyzing each transcript independently before engaging in cross-case analysis. Once all the interviews were completed, we developed Personal Experiential Themes (PETs) by grouping together experiential statements that reflected a similar idea or feeling within each interview. To do this, we compared notes, highlighted quotations, and exploratory comments from each transcript. We then looked for patterns in how participants described specific moments of Islamophobia and the meanings they attached to them.

After the PETs were created, we then compared them across interviews to see where participants described similar experiences and where their experiences differed. This comparison helped us identify which ideas were shared across the group and which were unique to particular individuals.

To create the final Group Experiential Themes (GETs), we first laid out all the PETs alongside the experiential statements and the key quotations that supported them and examined how they related to one another across participants. Visually seeing all the PETs, experiential statements and quotations in the same place helped us to understand which experiential themes or PETs were not truly reflective of the data, and new ones were created which were a better fit. Keeping the relevant quotations visible helped us ensure that the experiential statements and PETs reflected the participant's experience. Ultimately, the PETs which described similar experiences or reflected the same underlying meaning were grouped together, while PETs that represented distinct or contrasting experiences were kept separate. This process allowed us to see which patterns were consistent across participants and which reflected more individual experiences.

As we compared these clusters, identity emerged as a central thread running through the data. CRT helped deepen this interpretation by highlighting how racialized assumptions about intelligence, competence, and belonging shaped the meanings participants attached to their experiences. These insights guided the final organization of PETs into broader GETs that reflected how Muslim nurses were assigned identities, how they navigated these imposed identities, and how they ultimately reconciled them with their own sense of self. These GETs formed the final findings of the study.

## Ethical Considerations

Before recruiting and interviewing participants, ethics approval was obtained from the University of Calgary Conjoint Health Research Ethics Board on November 29, 2023. We removed any identifying information from the interview transcripts. The participant data was stored securely in line with organizational policy.

This study was guided by the recommendations outlined by Smith et al. (2022). In line with their guidance, the initial step of designing the study was shaped by the principle that IPA research should focus on how participants make sense of their lived experiences. This emphasis ensured that the study was structured to capture participants’ personal interpretations from the outset.

Next, Smith et al. (2022) suggest that the participant sample must be carefully selected to ensure it represents those with lived experience of the phenomena being studied. When choosing the inclusion criteria for this study, the study's goals were kept in mind.

The questions asked in the interviews were reflective of this study's purpose and reflective of Muslim nurses’ lived experiences with Islamophobia. The interview transcriptions were also reviewed to ensure accuracy. According to Smith et al. (2022), the researcher should keep a detailed account of the data analysis process. This was ensured by saving the files used at each step of the research process in a secure OneDrive folder.

## Findings

A total of six participants were included in this study. Demographic data for these participants are presented in Table 1. The findings are also listed in Table 3.

**Table 3.** Findings.

Group Experiential Theme	Sub Theme
The hijab assigns a social and professional identity to Muslim nurses	The ‘othering’ of Muslim nurses based on their assigned identity
	Intelligence, competence and the lack of respect for Muslim nurses
	The lack of support for Muslim nurses
	The impact of Islamophobia on Muslim nurses
Identity formation: How Muslim nurses question assigned identities and discover for themselves what it means to a Muslim nurse	Seeking a sense of belonging and acceptance with colleagues
Reconciling identities	How Muslim nurses handle situations where identities are assigned to them
	Finding community through connecting with others

The main findings of this study are that the hijab plays a significant role in prejudice towards Muslim Nurses and that racism and discrimination impact Muslim Nurses continuously from when they enter nursing school through to their workplaces. Also, Muslim nurses must be provided further support to be able to manage discrimination effectively.

Although the focus is on the experiences of Islamophobia faced by RNs in Canada in the healthcare setting, the interviews revealed that the racism and discrimination experienced by these nurses are not only limited to healthcare settings but reflect the systemic racism present in Canadian society.

## Theme 1- how the Hijab Assigns an Identity to Muslim Nurses Leading to Them Being Othered

Participants wearing the hijab became visually identifiable as being Muslim and were viewed through a stereotyped social identity of what it meant to be a Muslim. This one facet of their identity eclipsed all others and led to others projecting their assumptions about Islam onto these nurses. One participant shared what her manager said to her while working together: “*All the Black and Muslim people, they don't have good education, they don't know how to run a good livelihood. They end up in homelessness, poverty, beggars,…admit it that is a fact!… that's a reality*” (P.5). This participant's experience demonstrates how non-Muslims assigned harmful assumptions to Muslim nurses because they wear the hijab.

Another participant shared how she felt that wearing the hijab led to her being assigned a social identity, she said: “*Once I took my hijab off…people were more curious about getting to know me for who I am, the questions were pertaining to my role as a nurse rather than to my role as a Muslim woman”* (P.6)*.* This quotation demonstrates how a social identity is attached to Muslim nurses wearing hijab which is then used to view them through a particular lens. It also demonstrates how this perceived Muslim identity overtakes other identities of Muslim nurses who wear the hijab.

Another participant shared:

*He [a colleague] was reading the news, and it was about how someone in [country her family is from] passed away, a family member killed them, and he turns to me and [says]: I don't get how people believe in a religion that's so barbaric, alluding to Islam (*P.3).

By asking this, the colleague equated the actions of some Muslims with all Muslims, indirectly ‘othering’ the participant by placing her in a racialized category. In CRT, othering is a key concept that explains how marginalized groups are positioned as inherently different from the dominant group. This assigned identity marks Muslim nurses as distinct, which can lead to prejudiced and differential treatment in the workplace.

While discrimination based on skin colour is a form of racism, faith-based discrimination adds another layer of complexity. Black Muslim nurses in this study described experiencing bias not only due to race or gender but also because of their religion. Many participants are both Muslim and racialized, experiencing Islamophobia on multiple levels. One Black participant described feeling the compounded effects of being a Muslim nurse who is also Black, she shared…: “*Once I got into clinical … I had racist remarks. I've been called the N-word, I've been told to go back to my country, I've been called a terrorist”* (P.1). This participant, who was born in Canada, was assumed to be a foreigner, due to her skin colour and faith. Being referred to as a terrorist was also a reflection of how she was viewed as someone who shared violent ideologies with terrorists and must not be Canadian.

### Intelligence, Competence and the Lack of Respect for Muslim Nurses

One common assumption about Muslim women is that they are less intelligent and less competent than other nurses. While the previous section focused on assumptions about their faith, this section highlights assumptions about their professional competence as Registered Nurses. Participant 1 stated: “*People making assumptions that I wouldn't either be smart enough or that someone who looks like me can't …be practicing at a clinical level above someone who is a health care aide*” (P.1). Another participant shared a similar experience by stating: “*My clinical instructor [said]: people that look like you, they usually go down the health care aid route”* (P.4)*.* These experiences demonstrate how people with the nursing profession make these judgements about the intelligence and competence levels of Muslim Nurses.

Patients’ views of Muslim women were especially apparent when participants worked with other nurses and nursing students. Participant 3 in this study shared her experience training a new nursing student. She explained:

*I was training a student who was visibly Caucasian, and… some [patients] would direct all their questions towards her, despite me having years of experience more than her… I became the secondary person in the room …and her merit was automatically assumed*. (P.3)

This participant's experience highlights that, when standing next to a white woman ***,*** even a student ***,*** her knowledge as a Muslim nurse was automatically assumed to be inferior.

### The Lack of Support for Muslim Nurses

Muslim nurses in this study reported a lack of support when seeking help for Islamophobia or other workplace challenges. Participant 3 described raising her concerns with a manager, hoping for assistance. She shared:

*I shared all of these concerns …and she was just… very, superficially acknowledging what I said and at the end of it she [said]: you have a lot going on I hear you. Made it seem like it was issues that I was going through… essentially, she was dismissive…I was crying at one point because I was really hurt…she didn't acknowledge anything that I was saying*. (P.3)

By voicing her concerns, the nurse expected her manager to listen, acknowledge, and act. This quotation illustrates the manager's failure to recognize, investigate, or address the issue meaningfully.

Particpants in this study also spoke about the negative mental health impacts of Islamophobia. Participant 6 shared:

*You are taught patient safety, but we're worried about our safety too. With being a Muslim woman, there's an added layer to how do we protect ourselves…these are things that are on my mind…and it just gets exhausting over time and contributes to the burnout that we feel eventually*. (P.6)

Emotional exhaustion and feelings of burnout are common for nurses in Canada. Participant 6 described an additional layer of stress on top of being a nurse, due to being a Muslim woman. This added stress, due to feeling emotionally unsafe at her workplace, led to her constantly feeling that she needed to protect her professional identity.

The participants’ experiences highlight Islamophobia in Canada's nursing profession, particularly through the assignment of identity, which prompted them to reflect on how others labelled them and how they understood themselves in relation to the world.

### Identity Formation: how Muslim Nurses Question Assigned Identities and Discover for Themselves What it Means to be a Muslim Nurse

Participants in this study described how identities were assigned to them and how they resisted these labels to define what it means to be a Muslim nurse for themselves. In addition to being Muslim women, they also navigated multiple other identities, including nurse, student, professional, employee, wife, mother, daughter, and friend.

Participants spoke about the lack of representation of racialized women in the nursing profession and how they were made to feel different from others. Participant 2 shared: “*I did feel that I entered into a different environment when I entered. I didn't feel different, but when I was treated differently, that's when I felt, yes, I was different”* (P.2). This participant she came to this realization when she noticed the different treatment she received and was singled out due to being a Muslim.

Another participant described how having a social and professional identity attached to her prompted her to reflect on the values most important to her as a Muslim woman and as a human being. She stated: *I don't want to sacrifice my role as a Muslim woman for my role as a nurse or vice versa…they need to coexist* (P.6). She explained feeling forced to choose between her Muslim identity and her nursing identity. By removing her hijab, she blended in with other nurses on her unit and was more respected professionally. If she chose to maintain her Muslim identity by wearing the hijab, this led to more scrutiny from patients and less respect. The clash highlights the challenges faced by nurses who are visibly Muslim. Both of these identities relate to respect whereas removing the hijab gained her respect from others, but she risked losing self-respect.

### Seeking a Sense of Belonging and Acceptance with Colleagues

Given Muslim nurses’ diverse identities, participants have spoken about wanting to belong socially with their coworkers and about seeking a sense of belonging at the workplace. Participant 3 described this as follows: “*When I first started [my job]…I really wanted everyone to like me, and I wanted to make friends*” (P.3). This participant's experience describes how she tried to build a feeling of community with her coworkers and wanted to feel like she was part of the team.

The following participant's experience demonstrates how her Muslim identity acted as a barrier between her and other nurses. She shared: “*There were a few brown girls on my unit, they were very respected…they were not Muslim…but we all looked very similar. So I just felt maybe if I took off my hijab, I would start fitting in a bit more*” (P.6). This quotation demonstrates how this Muslim nurse had a strong and deep desire to belong with her colleagues and how she was willing to make an immense compromise, one which went against her core beliefs, just to be accepted by her colleagues.

### Finding Community Through Connecting with Others

This section examines how, when they felt socially isolated from coworkers, Muslim nurses sought ways to develop other social connections. One approach was connecting with other racialized women to debrief experiences of Islamophobia and gain emotional support.

Participant 4 describes how she found herself gravitating toward other racialized people in her search for acceptance. She described the process as follows: “*You find your own visible minority group, and you start looking out for each other and protecting each other because that's where you feel the most comfortable*” (P.4). The terms “looking out for” and “protecting” reflect this participant's sense that visible minorities in nursing lacked adequate support. Rather than relying on the organization, these women created informal communities to support one another, filling gaps left by their white colleagues.

Although participants in this study described many instances of Islamophobia, they also highlighted how some people made them feel supported. They described co-workers, faculty members, and nursing managers who were neither Muslim nor racialized people but still provided support to them. Participant 1 shared an example of this:

*I've* had many experiences where staff members [ask], are you okay? if they said something to you, I can do their care…and so that kind of accommodation was really helpful as an RN. (P.1)

Participant 1 expressed gratitude when her coworkers acknowledged the discrimination she faced, which led them to limit her interactions with abusive patients. These actions helped her feel more secure and fostered a sense of belonging and acceptance among her colleagues.

Although most participants reported negative and stereotyped experiences, Participant 3 noted that positive interactions were possible. She described how these experiences strengthened her connection with certain managers, saying:

*There are good managers out there and I think it really comes down to people that will assert themselves and understand that their role is not just to protect the public but to protect you know the people providing care to the public*. (P.3)

This quotation reflects Participant 3's experience of being supported by a manager who listened, believed her concerns, and took appropriate action. Notably, she felt that the manager also prioritized her well-being as an employee, not just patient care. This experience highlights that supporting nurses is essential for enabling them to provide high-quality care.

## Reconciling Identities

The final GET explores how Muslim nurses actively attempted to discover which parts of their identities they value and how this process helped them to find a middle ground, which led to them being more comfortable at work

Participant 1 who spoke about how, after having an identity assigned to her, she reflected on how she sees herself and her identity as a Black, Muslim woman. she shared:

*I also take so much pride in my identity, I'm so unique as a Black Muslim woman. There's not a lot that I can't be strong through. I know what I've been able to achieve being who I am, and I know that I can be strong and continue to achieve.* (P.1)

This participant's experience shows how she reframed being assigned an identity into actively defining her own, shaping how she presents herself in nursing. The quotation illustrates how she reflected on all her identities, including those imposed on her, to find strength in her unique position as a Black, Muslim nurse.

Participant 3 described embracing her Muslim identity when it was challenged at work by emotionally distancing herself from those who imposed labels and ignored her other unique identities. She said:

*I essentially shifted from caring a lot about what people thought about me to not caring at all unless I saw that they were trying to make an effort to get to know me for who I was, as opposed to what the media was depicting me as.* (P.3)

This participant described shifting her focus to building relationships with those who accepted her while disregarding the opinions of those who imposed an identity on her. By letting go of efforts to ‘fit in,’ she found greater peace and accepted the discomfort of not being fully accepted, prioritizing authenticity in expressing her Muslim identity.

Reconciling identities was an active, ongoing process, rather than a single event. Participant 6 in this study shared how, after removing her hijab at work, she chose to wear it again. She said: “*Returning back to covering my hair has been a lot of self-compassion and also a lot of pride in myself…*” (P.6). This participant's experience shows that, through introspection, she recognized that while wearing the hijab led to an assigned identity, her Muslim identity was essential to her sense of self. She decided to accept the assigned identity, as going to work without the hijab caused emotional distress. She shared:

*Both of your identities and both of your roles can coexist. You just need to find a way to do that…. I don't want it to feel like the two roles are conflicting; I want to keep both of them*. (P.6)

Although her work situation did not change or improve, this process of self-discovery allowed her to be able to be at peace with her identity as a Muslim woman and a nurse.

## Discussion

This study's findings demonstrate that Islamophobia is present across Canada's nursing profession, including acute care, community and mental health. By wearing the hijab, Muslim nurses are marked with a social and professional identity that carries assumptions about their faith, including perceptions of being supporters of terrorism, less intelligent, less competent and lacking agency. These assumptions contribute to Muslim nurses feeling othered, which negatively affects their personal and professional lives. Overall, the findings suggest that Islamophobia in Canadian nursing, through assigning a social identity to Muslim nurses, may harm the emotional, social, and professional well-being of Muslim nurses.

These findings align with key principles of CRT, particularly the process of racialization. As Garner and Selod (2015) argue, Muslims are often racialized through cultural and religious markers, such as the hijab, rather than through skin colour alone. Participants in this study described a similar process, with the hijab serving as a visible marker that triggered assumptions about their competence, values, and identity. This is consistent with Saleh et al. (2022), who found that hijab-wearing Muslim nurses are frequently viewed through stereotypes that undermine their professional credibility. These assumptions exemplify CRT's tenet of social construction, as they attribute traits to Muslim women that have no biological or genetic basis yet are treated as inherent characteristics of the group.

At the same time, our findings illustrate how racialization occurs not only in patient encounters but also through interactions with peers, and managers. These narratives reflect the ordinariness tenet of CRT by challenging dominant assumptions that racism in nursing is rare or exceptional. In doing so, this study contributes to the broader literature demonstrating that Islamophobia in healthcare is structural, embedded in institutional norms, and reproduced through everyday professional interactions.

The findings also reflect central concepts in IPA. Consistent with IPA's focus on how individuals make sense of significant life experiences (Smith et al., 2022), participants described not only what happened to them but also how they interpreted those events in relation to their identity as Muslim women and nurses. These results are similar to Rahmath et al. (2016), who found that hijab-wearing women often engaged in deep reflection about belonging and self-presentation. Participants in this study described an active process of questioning, negotiating, and redefining their identities in response to Islamophobia. This meaning-making reflects the phenomenological notion that identity is continually shaped through lived experience rather than being fixed or predetermined. In this way, the study expands IPA scholarship by illustrating how Muslim nurses interpret and integrate experiences of Islamophobia into broader, ongoing processes of self-understanding.

## How the Hijab Assigns an Identity to Muslim Nurses

The experience of Islamophobia often begins with being assigned the identity of a Muslim woman through wearing the hijab. This aligns with Saleh et al. (2022), who found that nurses wearing the hijab were subjected to stereotypes. Similar to Rahmath et al. (2016), this study found that women experienced discrimination after choosing to wear the hijab, which had not been present beforehand. The hijab thus serves as a visible marker of Muslim identity, making these women vulnerable to faith-based discrimination and Islamophobia (Environics Institute, 2016).

### The Relationship Between the Questioning of Intelligence, Competence and Leadership

A common misconception about the hijab is that Muslim women are forced to wear it and lack agency over their own lives. Nagra (2018) found that women wearing the hijab are viewed as being incapable of independent thought or decision-making. Similarly, Mahmud and Swami (2010) reported that British men perceive hijab-wearing women as less intelligent. According to Nagra (2018), Muslim women are often presumed to be passive, oppressed, uneducated, and unintelligent. The results of this study highlight that such assumptions are also made about Muslim nurses and can negatively affect them professionally, as colleagues and patients may question their ability to provide adequate care.

While this study focuses on Muslim nurses, it is essential to note that experiences of Islamophobia vary. The findings of this study show that Black Muslim nurses face compounded discrimination, experiencing both Islamophobia and racism. Similarly, Saleh (2001) found that Black Muslim nurses are subjected to multiple forms of discrimination, including biases related to both their race and their faith.

#### The Impact of Islamophobia on Muslim Nurses

Experiencing Islamophobia can take a significant emotional toll on Muslim nurses. Being othered positions them as social outcasts, with values, beliefs, and behaviours perceived as different from those of other nurses. This aligns with French (2022), who found that Muslim students felt socially isolated and misjudged by their peers. Social inclusion is so vital that the World Health Organization considers it a determinant of health (Marmot et al., 2008). Participants in this study described how social isolation negatively affected their mental health, echoing Pullen et al. (2023), who found that racialized workers, including Black staff, were more socially isolated at work, lacking both social ties and colleagues they could rely on for support.

After being assigned an identity of what it means to be a Muslim woman, participants began the work of identifying what being a nurse means to them along with being a Muslim woman. Meetoo and Mirza (2007) argue that Muslim women find themselves in the middle of multiple discourses, which work to define them, such as being oppressed by patriarchal figures and being caught between cultures. Participants in this study reported feeling as if their identities were imposed on them by others who expect them to believe and behave in particular ways. These findings are in alignment with the work by Latif (2024), who found that Muslim women perform and manage their identities in an environment in which they are discriminated against and stigmatized.

Participants in this study described seeking a sense of belonging by building closer social bonds with colleagues who could relate to and support their experiences. Portes and Rumbaut (2014) describe this as ‘reactive ethnicity,’ where marginalized individuals strengthen ties to their community when facing discrimination. Similarly, the nurses in this study also described how, upon experiencing Islamophobia, they strengthened their social connections with other Muslim women. Nagra (2017) notes that individuals often assert their personal agency when they feel their faith is being threatened.

Participants described reaffirming the values that are essential to them and discovering that they have multiple identities that can coexist. The study by MacDonald (2015) found that even though Muslims have strong connections with each other, they also find themselves identifying with an overall identity with those who are non-Muslim while feeling that their multiple identities can co-exist.

## Limitations

A limitation of this study is that it focuses only on RNs, not other types of nurses. This decision was made because training and workplace experiences can vary significantly across different nursing roles. Some units are exclusive to RNs or nurse practitioners, so limiting the sample to one type of nurse helped ensure consistency in participants’ educational and professional contexts.

Another limitation is that recruitment through social media may also have introduced self-selection bias, favouring nurses who felt strongly about this topic or who were already engaged in conversations about Islamophobia. While the sample reflects variation in age, work settings, and racial backgrounds, the study did not capture the full breadth of demographic diversity among Muslim nurses across Canada.

In IPA research, a small sample of five participants is considered sufficient, as the focus is on exploring each case in depth before comparing them (Eatough & Smith, 2017). Our study included six participants, consistent with IPA's emphasis on depth over breadth, allowing for detailed exploration of their experiences. While the small sample may limit the transferability of findings to the broader population of Muslim nurses, future studies with larger samples could build on these insights and examine experiences of Islamophobia across different nursing contexts. Despite this limitation, the study provides valuable insights into how Muslim nurses who wear the hijab experience, interpret, and navigate Islamophobia in the Canadian nursing profession.

## Recommendations for Practice

The findings of this study have several implications for nursing practice, administration, and education. First, nursing managers and leaders should recognize the emotional, social, and professional impacts of Islamophobia on Muslim nurses, particularly those who wear the hijab. Creating supportive and inclusive work environments, including precise reporting mechanisms for discrimination, mentorship programs, and peer support networks, can help mitigate the adverse effects of Islamophobia and foster a sense of belonging.

In terms of nursing education, curricula should incorporate content on cultural competence, anti-racism, and the racialization of religious identities to prepare students for diverse clinical environments. This education can help reduce biases and improve patient care by promoting understanding and respect for the multiple identities nurses may hold.

From a research perspective, future studies could build on these findings by exploring the experiences of Muslim nurses in different roles, settings, or regions, or by examining interventions designed to reduce Islamophobia in the workplace. Investigating how systemic policies and organizational practices influence the well-being and retention of racialized nurses could further strengthen evidence-based strategies for equity in nursing.

Overall, these recommendations provide practical guidance for improving nursing practice and education while highlighting areas where further research is needed to support Muslim nurses and promote inclusive healthcare environments.

## Conclusion

This study demonstrates that Islamophobia is a persistent and multifaceted challenge within Canadian nursing, particularly for female Muslim nurses who wear the hijab. Being visibly identified as Muslim often leads to the assignment of stereotypes and othering, which can undermine perceptions of competence, limit opportunities for professional growth, and create social and emotional strain. Participants’ experiences illustrate how these nurses navigate complex intersections of faith, gender, and race, actively reconciling their multiple identities to maintain professional and personal integrity.

The findings underscore the urgent need for nursing workplaces and educational environments to implement structural and practical interventions. These include clear anti-discrimination policies, inclusive workplace practices, culturally competent leadership, mental health support, and peer support networks to ensure that Muslim nurses feel valued, safe, and supported. Addressing Islamophobia is not only an issue of equity but also essential for fostering collaborative teams and high-quality patient care.

For future research, this study highlights the importance of exploring Islamophobia across different nursing roles, examining intersectional impacts of race, gender, and religion, and evaluating interventions designed to reduce discrimination and promote inclusion. By illuminating the experiences of Muslim nurses, this study provides a foundation for policy and practice changes to create a more equitable, supportive, and resilient nursing workforce in Canada.

## Appendix A: Interview Guide

### Interview Guide

I am planning to interview 8 participants across Canada who identify as female, Muslim and are currently employed as Registered Nurses. In these interviews, I hope to gain further insight into their experiences of Islamophobia in the nursing profession. The results of this study will help to guide future research on nurses who experience Islamophobia in Canada.

Interviews will be conversational and open-ended; however, this interview guide will give some structure to the conversation. I will ask follow-up questions, such as ‘Can you tell me more about what you meant when you said…?’ or ‘Can you expand on that?’

#### Introduction/Background

Welcome the participant.
Please tell me about how long you have been a Registered Nurse and your role at work.
Can you please tell me your year of birth?
Please let me know your racial and/or ethnic identity.

#### Experiences of Islamophobia

Can you tell me about a time when you faced discrimination as a Registered Nurse or nursing student in Canada? Follow up questions such as the following may be asked: How did you feel when this happened? How do you feel now that you have had a chance to reflect on this incident? What role were you practicing in when you experienced Islamophobia?
Did these experiences have any impact on your work as a nurse?
How does Islamophobia affect your professional role?

#### Closing

Thank the participant for their time and for sharing their valuable knowledge and experiences.
